# Mtu1 defects are correlated with reduced osteogenic differentiation

**DOI:** 10.1038/s41419-020-03345-5

**Published:** 2021-01-11

**Authors:** Qiufen He, Qiong Zhao, Qianqian Li, Ruolang Pan, Xiongfeng Li, Ye Chen

**Affiliations:** 1 Division of Medical Genetics and Genomics, The Children’s Hospital, Zhejiang University School of Medicine, Hangzhou, Zhejiang 310058 China; 2 Institute of Genetics, Zhejiang University and Department of Genetics, Zhejiang University School of Medicine, Hangzhou, Zhejiang 310058 China; 3 Zhejiang Provincial Key Laboratory of Genetic and Developmental Disorders, Hangzhou, Zhejiang 310058 China; 4 Institute for Cell‐Based Drug Development of Zhejiang Province, S‐Evans Biosciences, Hangzhou, Zhejiang 310012 China; 5 Key Laboratory of Cell‐Based Drug and Applied Technology Development in Zhejiang Province, Hangzhou, Zhejiang 310058 China; 6 Department of Orthopedics, Huzhou Central Hospital, Zhejiang University Huzhou Hospital, Huzhou, Zhejiang 313000 China

**Keywords:** Cell biology, Post-translational modifications, Mesenchymal stem cells

## Abstract

Accumulating evidence has revealed that mitochondria dynamics and function regulation is essential for the successful mesenchymal stem cell (MSC) differentiation. In the present study, the researchers reported for the first time that Mtu1 defects are correlated with reduced osteogenic differentiation. Using in vitro cultured bone marrow MSCs and stromal cell line MS5, we demonstrated that depressed Mtu1 expression was associated with reduced 2-thiouridine modification of the U34 of mitochondrial tRNA^Gln^, tRNA^Glu^, and tRNA^Lys^, which led to respiratory deficiencies and reduced mitochondrial ATP production, and finally suppressed osteogenic differentiation. As expected, these Mtu1-deficient mice exhibited obvious osteopenia. Therefore, our findings in this study provide new insights into the pathophysiology of osteopenia.

## Introduction

Mesenchymal stem cells (MSCs), which are multipotent cells readily accessible from various tissues and give rise to mesoderm cell lineages such as osteoblasts and adipocytes, have been widely studied over the past two decades. Cell-based regenerative therapies using MSCs are emerging as promising therapeutic approaches in various diseases, such as bone and cartilage, cardiovascular, autoimmune, and liver diseases, and cancer^1–5^. However, the specific mechanisms regulating the fate of MSCs are still not fully understood.

In mammalian cells, mitochondria are the major energy source, which provide ATP through oxidative phosphorylation (OXPHOS), and also play a critical role in reactive oxygen species (ROS) production and programmed cell death (apoptosis)^6–8^. Recent studies have demonstrated the importance of mitochondrial metabolism in regulating stem cell biology. Like other stem cells, MSCs own a relatively low mitochondria activity until the start of cell differentiation^9,10^. Marked changes in mitochondrial DNA (mtDNA) copy number, protein levels of respiratory enzymes, oxygen consumption rates (OCRs), mRNA levels of mitochondrial biogenesis-associated genes, and intracellular ATP content were observed upon differentiation induction^11–14^. These studies indicate that mitochondria play an important regulatory role in differentiation capacity of MSCs^15^. Understanding the roles of mitochondrial dynamics during MSC differentiation will facilitate the optimization of differentiation protocols, and ultimately, benefit the development of new pharmacologic strategies in regenerative medicine.

Mitochondrial defects have been associated with a wide spectrum of degenerative diseases, aging, and cancer^16–18^. Previous studies demonstrate that global knockout mouse models of mitochondrial genes are heterozygous haploinsufficient and homozygous-lethal^19^. Furthermore, the abolished of OXPHOS resulted in embryonic lethality approximately at the time of organogenesis, suggesting the importance of mitochondria in cell differentiation^20–23^. More appropriate models are needed to better delineate the role of mitochondrial dysfunction during stem cell differentiation. In the present study, heterozygous Mtu1 (also known as Trmu, a mitochondrial-tRNA-modifying enzyme) knockout mice that exhibited symptoms of osteopenia were adopted as a potential model. This Mtu1 deficiency was further evaluated for its effects on mitochondrial translation, respiration, and ATP production using primary bone marrow MSCs and MS5 stromal cell line.

## Materials and Methods

### Animal model

C57BL/6 mice were purchased from Shanghai SLAC Animal Inc. (Shanghai, China). The Mtu1 knockout mice were generated using the CRISPR/Cas9 method. Briefly, C57BL/6 female mice were superovulated and mated with C57BL/6 males, and fertilized eggs were collected from the oviduct. The pronuclear stage eggs were injected with Cas9 mRNA and sgRNA. The eggs were cultivated in KSOM overnight then transferred into the oviducts of pseudopregnant ICR females. The sequence of sgRNA used for Mtu1 is 5′-CACGTCGTGTGCTCCCTGTC-3′. Genomic DNA was extracted from the tails, and a DNA fragment surrounding the target site was PCR-amplified with specific primers to identify the genotype of the offspring. The PCR primers are as follows: forward 5′-ACTTCCGGCGTAGCTTGGA-3′ and reverse 5′-ATGCGGAGAGGTTGACACAA-3′. Mice were housed on a 12-h light/dark cycle, and provided food and water ad libitum in the Experimental Animal Center of Zhejiang University. The research was approved by the Animal Ethic Committee of Zhejiang University (Ethical Approval Code: 17264). Experiments and animal care were performed in accordance with the guidelines of Zhejiang University.

### MS5 stromal cell line

MS5 stromal cell line, a type of mouse adherent fibroblastic cells growing in monolayers, was obtained from Leibniz Institute DSMZ-German Collection of Microorganisms and Cell Cultures GmbH. The cells were maintained in Dulbecco’s Modified Eagle Medium (DMEM, Hyclone) supplemented with 10% fetal bovine serum (FBS, Gemini) at 37 °C with 5% CO_2_.

### Knockdown of Mtu1 in MS5 stromal cells

The shRNAs containing a hairpin loop were synthesized and inserted into the pLKO.1-puro vector, and the sequences were shown in Supplementary Table S1. Lentiviral vectors, and pLKO.1 with shRNA were co-transfected into HEK293T cells with psPAX2 and pMD2.G for lentivirus production^24^. 2 days after infection, the transduced cells were selected using 1 µg/mL puromycin over a two-week period, resulting in homogeneous populations.

### Cell isolation and culture of mouse BM-MSCs

BM-MSCs were prepared as previously described^25^. Femurs and tibias from wild type and *Mtu1*^*+/−*^ mice (four-month-old) were dissected and flushed with PBS. The bones were chopped and incubated for 1.5 h at 37 °C in 10 mL of DMEM containing 1 mg/mL collagenase II. The pellet was collected by centrifugation at 800 g for 4 min and cultured in Minimum Essential Medium Eagle - Alpha Modification (Alpha MEM, Hyclone) supplemented with 10% FBS and 1% penicillin/streptomycin at 37 °C with 5% CO_2_. After 3 days, nonadherent cells and debris were removed and the adherent cells were continuously cultured.

### MSC Characterization

Cells were trypsinized, washed in PBS, and stained with the following anti-mouse antibodies: CD44-phycoerythrin (PE; cat:130-102-606), CD45-PE (cat:130-102-781), F4/80-fluorescein isothiocyanate (cat:130-102-327), and Sca-1-allophycocyanin (cat:130-102-343) from Miltenyi Biotec. The samples were processed using a NovoCyte flow cytometer (ACEA Biosciences) and analyzed using the NovoExpress software.

### Western blot analysis

Here, 20 µg of total cellular proteins obtained from MSCs were denatured and loaded on sodium dodecyl sulfate polyacrylamide gels. After electrophoresis, the gels were transferred to a PVDF membrane (Millipore) and processed for immunoblotting. Commercially available antibodies, such as MTU1 (ab50895), ND5 (ab92624), NDUFB8 (ab110242), SDHB (ab14714), UQCRC2 (ab14745), ATP5a (ab14748), and MTCO1 (ab17405) from Abcam, ATP6 (55313-1-AP), GAPDH (60004-1-Ig), and VDAC (55259-1-AP) from Proteintech, NDUFS1 (A16926), NDUFS2 (A12858), ND4 (A17970), and CYTB (A17966) from Abclonal were used. Peroxidase AffiniPure Goat Anti-Mouse IgG and Goat Anti-Rabbit IgG (Jackson) were used as secondary antibodies, and the protein signals were detected using the ECL system. Band intensities were quantified from the 16-bit digital image by densitometry in ImageJ and are shown normalized to GAPDH for each target.

### Quantitative real-time PCR

The extracted total RNAs were reverse-transcribed into complementary DNA using the PrimeScript™ RT Reagent Kit (Takara), and quantitative real-time PCR analyses were performed using an Applied Biosystems Prism 7900 System with gene-specific primers. Data were normalized to the mRNA levels of Gapdh that was used as a housekeeping gene and were analyzed using the 2^−ΔΔCT^ method. Detailed primer sequences are provided in Supplementary Table S1.

### Bone structure analysis

To evaluated the bone mineral density (BMD) and microarchitecture of femur trabeculae in mice, male mice at 6 weeks, 12 weeks, and 48 weeks ages were scanned in a U-CT system (MILabs U-CT, Netherlands). Datasets were reconstructed using MILabs Rec 10.16 software. Trabecular BMD, BS/BV, BV/TV, Tb.Th, and Tb.Sp were calculated from the region of interest (ROI). The *μ*CT data were evaluated and three-dimensional bone structure image slices were reconstructed using the IMALYTICS Preclinical 2.1 software.

### Transcriptome-seq and bioinformatic analysis

The total RNAs were isolated using TRIzol reagent (Ambion Inc.) following the manufacturer’s procedure. The total RNA quantity and purity were analysis of Bioanalyzer 2100 and RNA 6000 Nano Lab Chip Kit (Agilent, CA, USA) with RIN number >7.0. cDNA libraries were generated using NEB Next Ultra Directional RNA Library Prep Kit (NEB), and then sequenced using the Illumina sequencing technology on an Illumina Hiseq 4000 at LC Bio (Zhejiang, China) according to the recommended protocols. Paired-end clean reads were mapped to the mouse reference genome GRCm38/mm10 using TopHat software^26^. The genome-matching reads were used to measure mRNA abundance using Cufflinks software^27^. The mRNAs at a cut-off of 10 reads were compared and considered as differentially expressed if the fold change > 2 and *P* value < 0.05. Gene set enrichment analysis (GSEA) was performed by inputting a list in which differentially expressed genes were ranked according to their fold change, into GSEA application^28^. KEGG pathway analysis was performed using Cluster Profiler R package. The RNA sequencing (RNA-Seq) data were deposited into Sequence Read Archive (SRA) with the Bioproject ID of PRJNA670203.

### Statistical analysis

Statistical analysis was carried out based on at least three independent experiments using unpaired two-tailed Student’s t-test (or ANOVA) in the GraphPad Prism seven program. The error bars indicate two standard deviations of the means. Differences were considered significant at a *P* value of <0.05.

Other detailed methods are available in the Supplementary Material and Methods online.

## Results

### Osteopenia induced in Mtu1-deficient mice

**Fig. 1:**
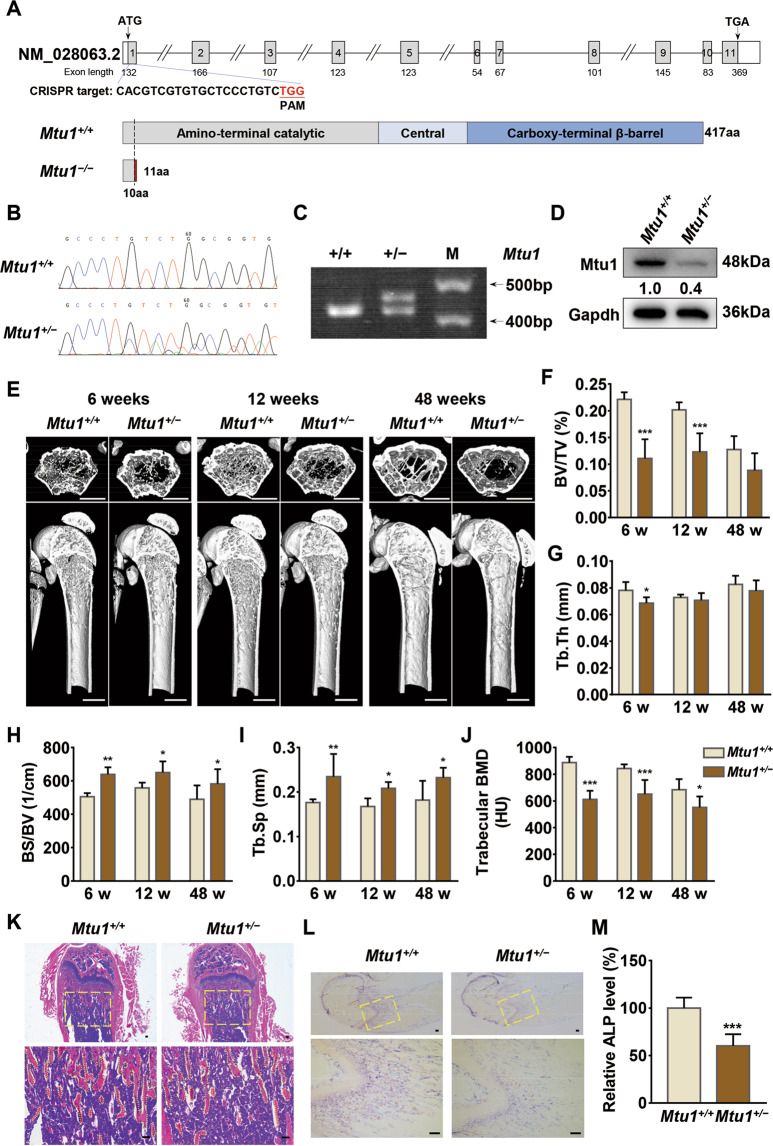
Decreased bone mass in Mtu1 knockout mice. **A** Schematic representation of CRISPR/Cas9 target site at exon 1 of *Mtu1* gene, as used in this study. The resultant truncated 11 aa non-functional protein caused by frameshifting insertion in Mtu1 is shown in the diagram. **B**, **C** Genotyping of Mtu1 knockout mice by Sanger sequencing and agarose gel electrophoresis. The PCR product size for genotyping is 417 bp in *Mtu1*^*+/+*^ mice and 417 bp/452 bp in *Mtu1*^*+/−*^ mice. **D** Western blot analyses show that BM-MSCs isolated from *Mtu1*^*+/−*^ have lower Mtu1 expression levels. **E** *μ*CT images of the femurs of wild-type and Mtu1 deficient male mice at 6 weeks, 12 weeks, and 48 weeks. Scale bars: 1 mm. In the analysis of the trabecular bone and architecture, the following parameters were calculated: **F** Bone volume per tissue volume (BV/TV); **G** Trabecular thickness (Tb.Th); **H** Bone surface to bone volume (BS/BV); **I** Trabecular spacing (Tb.Sp); **J** Trabecular bone mineral density (BMD). *n* = 6 per group. The region of interests (ROI) for trabecular parameters calculation were chosen at the central of medullary cavity with cylinders (1 mm in diameter, 1 mm in height) at 1 mm away from epiphyseal line. **K** H&E staining and **L** ALP staining of distal femoral sections of 4 weeks old mice. Scale bars: 100 μm. **M** The relative levels of positive ALP staining area in the yellow dotted line at the femur were quantified using Image J. *n* = 5 per group. *P < 0.05; **P < 0.05; ***P < 0.001.

Researchers generated a 35-bp insertion, which caused a frameshift from codon 10 and then terminated early at codon 11, in the exon 1 of *Mtu1* knockout mice through CRISPR/Cas9 technology to test the role of Mtu1 in MSC differentiation (Fig. 1A). This allele was subsequently propagated after confirming the mutation by Sanger sequencing, agarose gel electrophoresis and Western blot analysis (Fig. 1B–D). There was no *Mtu1*^*−/−*^ mice obtained after multiple generations of breeding, which was consistent with previous research that *Mtu1*^*−/−*^ mice result in embryonic lethality around E7.5-8^29^.

Like the wild-type mice, *Mtu1*^*+/−*^ mice exhibited normal body size and lifespan. The researchers tested their femur health at different ages in a U-CT system (Fig. 1E). The results showed decreased bone volume per tissue volume (BV/TV; Fig. 1F), trabecular thickness (Tb.Th; Fig. 1G), as well as significant increased specific bond surface (BS/BV; Fig. 1H) and trabecular separation (Tb.Sp; Fig. 1I), indicating significant poor bone quality in *Mtu1*^*+/−*^ mice compared with wild type mice. In addition, the quality of Trabecular (bone mineral density; BMD; Fig. 1J) was also significantly lower in *Mtu1*^*+/−*^ mice. Given these observations from the *μ*CT analysis, we proposed that Mtu1 deficiency could contribute to bone loss and dysplasia, resulting in the pathophysiology of osteopenia. Furthermore, hematoxylin/eosin (H&E) staining of femur sections also showed a shortening of the trabecular bone in four-week-old *Mtu1*^*+/−*^ mice, compared with the *Mtu1*^*+/+*^ littermates (Fig. 1K). In the meanwhile, ALP, a sensitive and reliable indicator of bone metabolism reflecting biosynthetic activity of the bone-forming cells, was also found decreased in *Mtu1*^*+/−*^ mice femur sections (Fig. 1L, M). These findings indicated that Mtu1 mutant resulted in abnormal bone development in mice.

### Generation of BM-MSCs from Mtu1 knockout mice

**Fig. 2:**
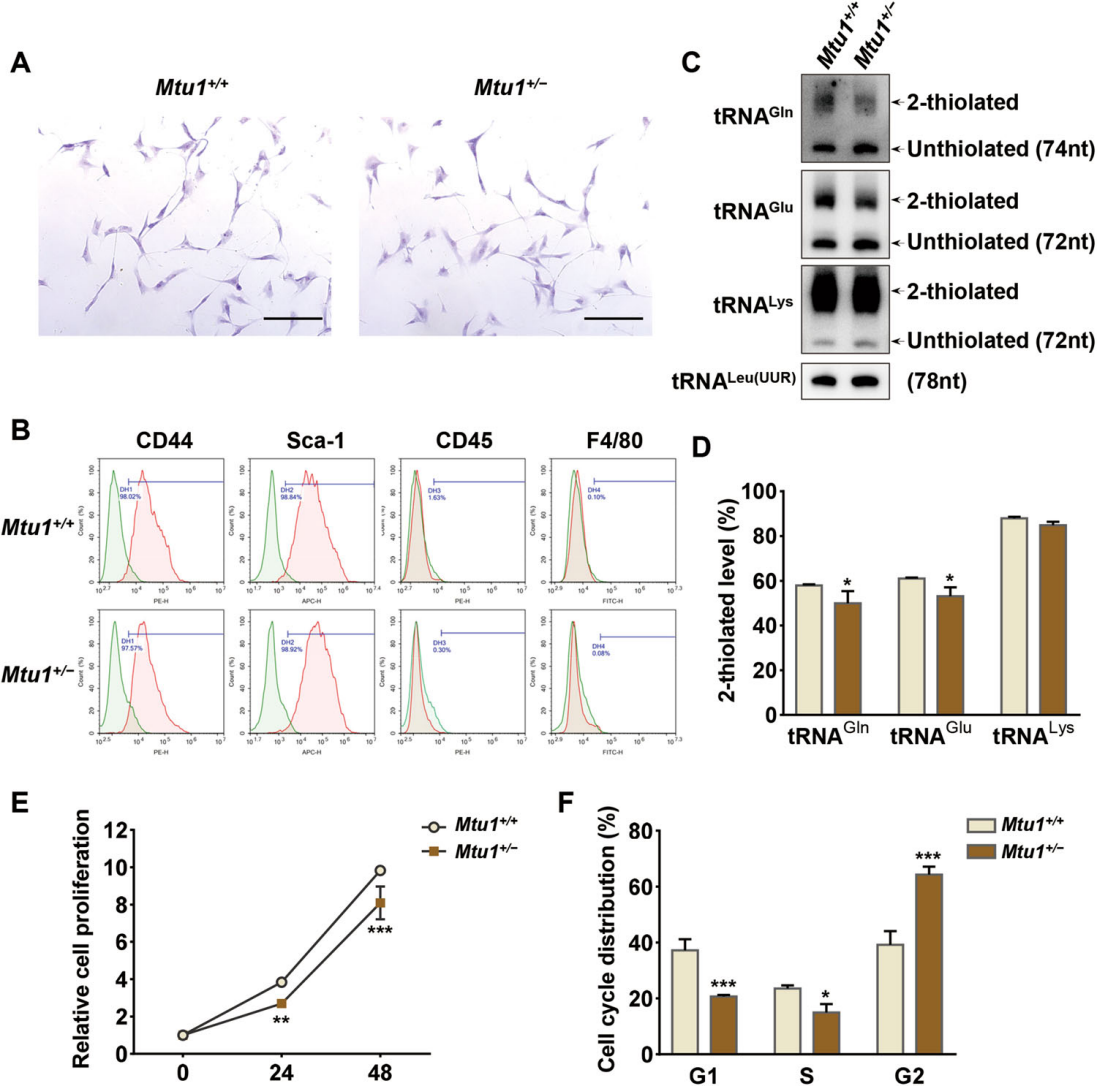
*Mtu1*^*+/−*^ BM-MSCs had reduced 2-thiouridylation levels of mitochondrial tRNAs. **A** Crystal violet staining of mouse BM-MSCs. Scale bars: 100 μm. **B** Representative results of flow cytometric analysis for cell-surface markers. Green peaks, isotype control; red peaks, specific antibodies. **C** 2-Thiouridylation level analysis of mt-tRNAs by APM gels. **D** Proportion of the 2-thiouridine levels of tRNAs. The proportion values are expressed as ratios (%) of the average of 2-thiouridine modified tRNA levels to total levels. **E** Cell proliferation assay. Cell counts were carried out on hours 0, 24, and 48 after seeding. **F** Cell cycle analysis. Flow cytometry results indicating the proportion of cells in the G1, S, and G2 phase. *P < 0.05; **P < 0.01; ***P < 0.001.

BM-MSCs were isolated from four-month-old mice to further investigate the influence of Mtu1 deficiency on MSC differentiation. These MSCs were purified by adherence to plastic and identified using expression of cell-surface markers, such as CD44 and Sca-1, and lack of expression of F4/80 and CD45 surface molecules^30,31^. As shown in Fig. 2A, the *Mtu1*^*+/−*^ MSCs displayed the same morphology as the *Mtu1*^*+/+*^ control, and flow cytometric analysis indicated that both genotypes contained a consistent subset surface marker (CD44^+^Sca-1^+^CD45^−^F4/80^−^) (Fig. 2B).

The BM-MSCs isolated from *Mtu1*^*+/−*^ mice showed a significantly reduced level of Mtu1 (60% relative to the wild-type, Fig. 1D). The researchers then evaluated the 2-thiouridylation levels of tRNA^Gln^, tRNA^Glu^, and tRNA^Lys^ through electrophoresis mobility retardation in polyacrylamide gel containing 0.05 mg/ml ((N-acryloylamino)phenyl) mercuric chloride (APM)^32,33^, with tRNA^Leu(UUR)^ as the negative control. In this system, the upper band represented the thiouridylated tRNA, and the lower band was unthiouridylated tRNA. As shown in Fig. 2C, D, the 2-thiouridylation levels of tRNA^Gln^, tRNA^Glu^, and tRNA^Lys^ were reduced in *Mtu1*^*+/−*^ BM-MSCs, compared with wild type BM-MSCs. The 2-thiouridylation proportions of tRNA^Gln^, tRNA^Glu^, and tRNA^Lys^ in *Mtu1*^*+/−*^ BM-MSCs were 49.9%, 53.1%, and 84.8%, respectively, whereas those in wild-type BM-MSCs were 58.0%, 61.0%, and 88.0%, respectively. In addition, the downregulation of cellular proliferation and G2 phase cell cycle arrest suggested that *Mtu1*^*+/−*^ BM-MSCs growth was repressed (Fig. 2E, F). These results indicated that *Mtu1*^*+/−*^ mice did affect the 2-thiouridylation levels of mitochondrial tRNAs and slow the cell growth but not the basal characteristics of MSCs, such as morphology and cell-surface markers.

### Reduced osteogenic differentiation capacity in *Mtu1*^*+/−*^ BM-MSCs

**Fig. 3:**
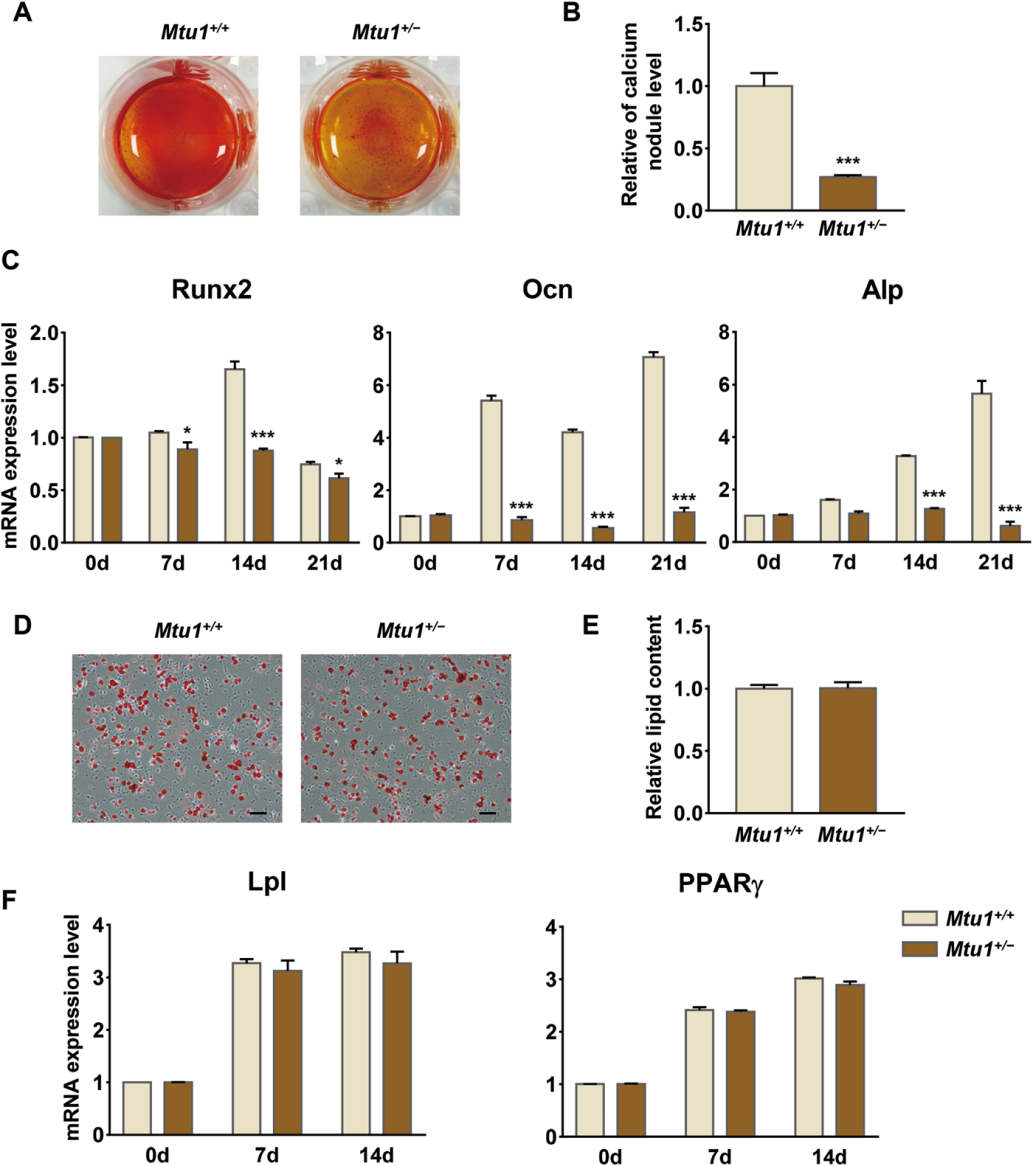
BM-MSCs from *Mtu1*^*+/−*^ affected osteogenic, but not adipogenic, differentiation. **A**, **B** Alizarin Red S staining and quantification of mineralized calcium deposits in cells after 14 days of osteogenic differentiation. The figures show one representative result of at least three experiments. **C** RT-qPCR at 0, 7, 14, and 21 days for mRNA levels of osteoblast differentiation markers, including *Runx2*, *Ocn*, and *Alp*. **D**, **E** Oil Red O staining and quantification of accumulated lipid droplets in cells after 14 days of adipogenic differentiation. The figures show one representative result of at least three experiments. Scale bars: 100 μm. **F** RT-qPCR at 0, 7, and 14 days for mRNA levels of adipocyte differentiation markers including *Lpl* and *PPARγ*. Scale bars: 100 μm; *P < 0.05; ***P < 0.001.

Osteogenic and adipogenic differentiation were induced to verify whether Mtu1 defects affected MSCs in terms of giving rise to lineage cells. The Alizarin Red S staining manifested that the intensity of mineralized calcium was decreased in *Mtu1*^*+/−*^ after 14 days of osteogenic differentiation (Fig. 3A), and the quantitative analysis of calcium nodule levels evidenced a statistically significantly reduced by 73.1% (Fig. 3B). In addition, qPCR analysis also revealed decreased osteoblast markers (*Runx2*, *Ocn*, and *Alp*) in mutant cells during osteogenic differentiation (Fig. 3C).

Moreover, at the end of adipogenic differentiation, on day 14, Oil Red O staining was performed to visualize the accumulated lipid droplets in differentiated adipocytes. The result showed that there was no significant change of lipid accumulation in *Mtu1*^*+/−*^, compared with the wild-type (Fig. 3D, E), which was confirmed by assessing adipogenic marker gene expression (*Lpl* and *PPARγ*) (Fig. 3F).

### Mitochondrial dysfunctions in *Mtu1*^*+/−*^ BM-MSCs

**Fig. 4:**
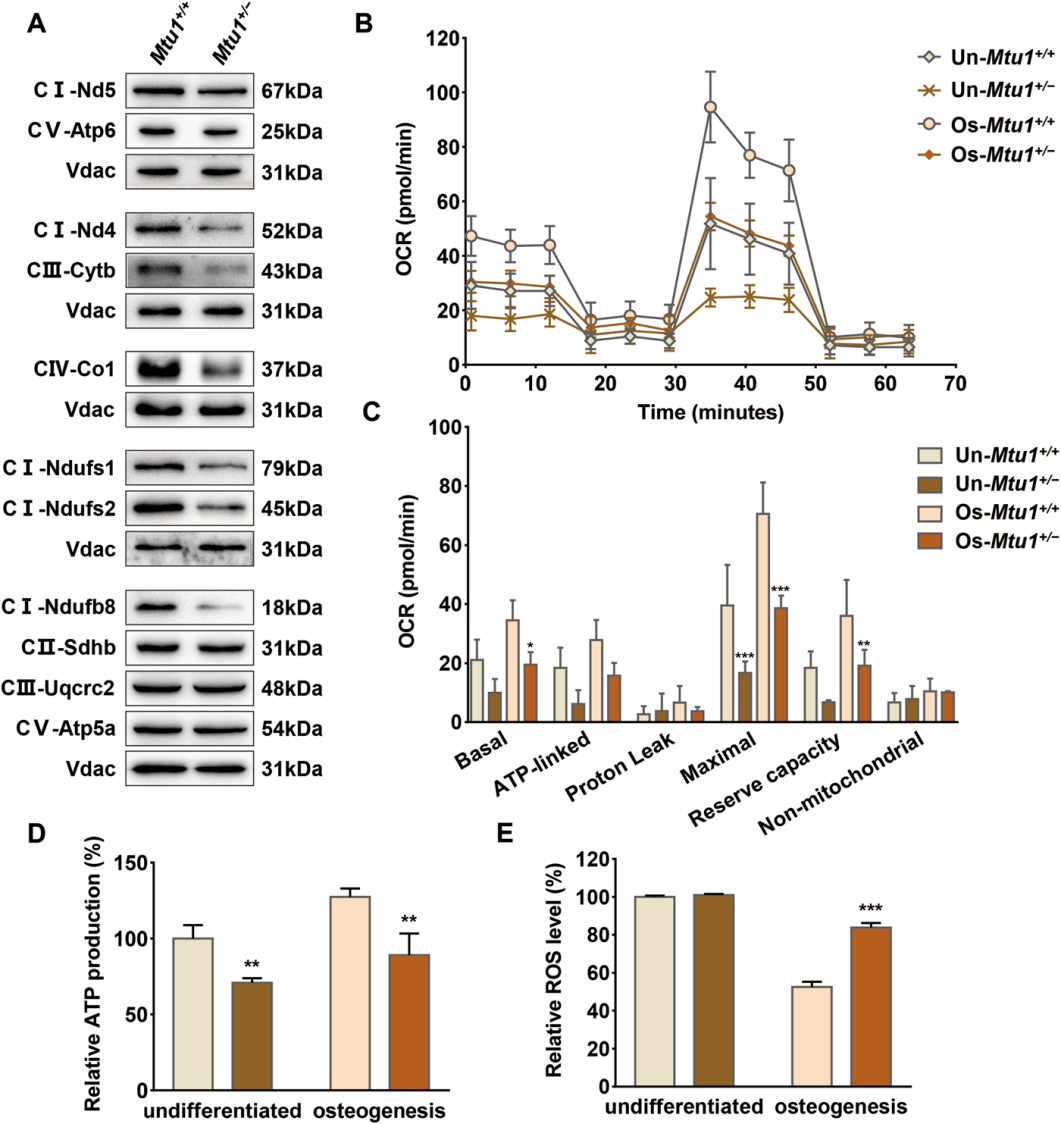
Mitochondrial dysfunction was elevated in undifferentiated and osteogenic cells. **A** Western blot analysis of OXPHOS complexes subunits (CI~CV), with Vdac as a loading control. **B** Analysis of OCRs in undifferentiated and osteogenesis cell lines using multiple inhibitors. **C** Quantification of various OCRs revealed significant respiration deficiency. The figure presented the ATP-linked OCR, proton leak OCR, maximal OCR, reserve capacity, and non-mitochondrial OCR in various cell lines. **D**, **E** Relative mitochondrial ATP and ROS production in various cell lines. *P < 0.05; **P < 0.01; ***P < 0.001.

Previous studies have shown that the 2-thiouridylated modification reduction caused by Mtu1 defects can affect mitochondrial protein translation^29,34,35^. 13 subunits encoded by mtDNA and >70 subunits encoded by the nuclear genome together form the OXPHOS complexes (CI~CV). The researchers typically examined the levels of five mtDNA-encoding polypeptides (Nd4, Nd5, Cytb, Co1 and Atp6) and six nDNA-encoding polypeptides (Ndufs1, Ndufs2, Ndufb8, Sdhb, Uqcrc2, and Atp5a) through Western blotting with Vdac as a loading control, to investigate whether the Mtu1 knockout impaired mitochondrial OXPHOS complexes in MSCs. Notably, the protein levels of Nd4, Ndufs1, Ndufs2, and Ndufb8 (CI) as well as Cytb (CIII), Co1 (CIV) showed an evident reduction in *Mtu1*^*+/−*^ BM-MSCs compared with the control group (Fig. 4A). It was also found that Nd5 and Atp6 remained at lower levels in Mtu1^+/−^ BM-MSCs.

Generally, in adipogenesis and osteogenesis differentiated cells, increased mitochondrial biogenesis was observed, increasing abundance of mitochondria^15^. Here, the researchers determined the OCR of undifferentiated and osteogenesis cells. For BM-MSCs from wild type mice, the energetics analysis showed that the basal, ATP-linked, and maximal respiration, as well as reserve capacity were increased after osteogenesis differentiation (Fig. 4B). Both undifferentiated and osteogenesis *Mtu1*^*+/−*^ BM-MSCs had reduced basal, ATP-linked, maximal respiration, and reserve capacity (Fig. 4C). Moreover, the results of the ATP production levels in undifferentiated and osteogenesis cells were consistent with those of the OCR assay (Fig. 4D). Furthermore, the mitochondrial ROS production was decreased after osteogenesis differentiation, and *Mtu1*^*+/−*^ BM-MSCs showed increased mitochondrial ROS production in osteogenesis cells, compared with the control group (Fig. 4E). These results demonstrated that Mtu1 deficiency leads to impaired mitochondrial translation and respiratory activities both in undifferentiated and osteogenesis BM-MSCs, and the impairments were magnified in osteogenic differentiation.

### Knockdown of Mtu1 in MS5

**Fig. 5:**
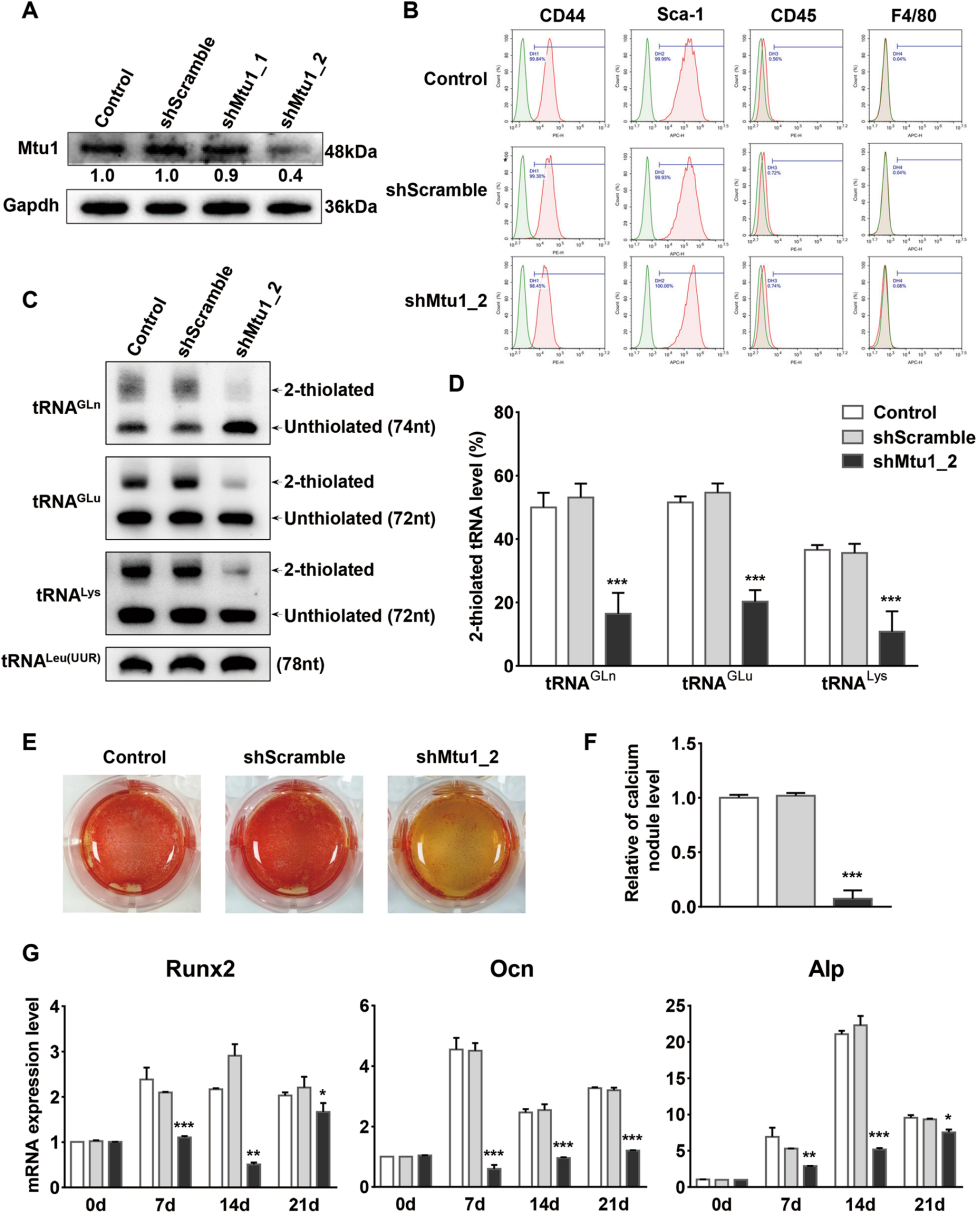
Mtu1 knockdown reduced the mitochondrial tRNA 2-thiouridylation level in MS5 stromal cells. **A** Mtu1 was silenced in MS5 stromal cells using distinct Mtu1 shRNA-encoding lentivirus or scramble lentivirus. Western blot analyses show lower Mtu1 expression levels in shMtu1_2 cells. **B** Flow cytometric analysis for cell-surface markers. Green peaks, isotype control; red peaks, specific antibodies. **C** 2-Thiouridylation level analysis of mt-tRNAs by APM gel. **D** Proportion of tRNA 2-thiouridine modification levels. The proportion values are expressed as ratios (%) of the average of 2-thiouridine modified tRNA levels to total levels. **E**, **F** Alizarin Red S staining and quantification of mineralized calcium deposits in cells after 14 days of osteogenic differentiation. The figures show one representative result of at least three experiments. **G** RT-qPCR at 0, 7, 14, and 21 days for mRNA levels of osteoblast differentiation markers, including *Runx2*, *Ocn*, and *Alp*. *P < 0.05; **P < 0.01; ***P < 0.001.

To further explore the effects of Mtu1 on osteogenesis differentiation, the MS5 stromal cell line was utilized for the shRNA-mediated Mtu1-knockdown experiment. The Mtu1 level in the resultant stable cell lines was examined by Western blotting, and the shMtu1_2 cell line demonstrated a significant decrease of Mtu1 expression level compared with control and shScramble cell lines (Fig. 5A). Flow cytometric analysis indicated that both shScramble and shMtu1_2 cells contained a consistent subset surface marker (CD44^+^Sca-1^+^CD45^−^F4/80^−^) similar to control cells (Fig. 5B). Apart from this, the cell morphology of Mtu1 knockdown did not significantly change (data not shown). The APM gel analysis showed a significant reduction in the 2-thiouridylation levels of tRNA^Gln^, tRNA^Glu^, and tRNA^Lys^ in the Mtu1 knockdown cell lines, as compared with those in control and shScramble groups (Fig. 5C). The proportions of the 2-thiouridylation levels of tRNA^Gln^, tRNA^Glu^, and tRNA^Lys^ in Mtu1 knockdown cell lines were 16.4%, 20.2%, and 10.8%, respectively, whereas those in the scrambled cell lines were 53.1%, 54.6%, and 35.6%, and they were 50.0%, 51.5%, and 36.5%, respectively, in the control cell lines (Fig. 5D).

Similarly, osteogenic and adipogenic differentiation were performed for control, shScramble, and shMtu1_2 cell lines, respectively. The shMtu1_2 cells manifested a 92.5% reduction of mineralized calcium intensity after 14 days of osteogenic differentiation compared with the control group (Fig. 5E, F) and also revealed decreased osteoblast markers *Runx2*, *Ocn* and *Alp* during osteogenic differentiation (Fig. 5G). Furthermore, lipid accumulation and adipogenic marker gene expression (*Lpl* and *PPARγ*) were indistinguishable among control, shScramble, and shMtu1_2 cells (Supplementary Fig. S1).

Accordingly, the mitochondrial respiratory chain associated polypeptides were impaired in shMtu1_2 cells, which was consistent with these findings in *Mtu1*^*+/−*^ BM-MSCs (Supplementary Fig. S2A). A broad and significant decrease in the activities of Complexes I, III, and IV resulted from disruption of mitochondrial translation and the activities of Complexes I, II, III, and IV in shMtu1_2 cell lines were 43.6%, 92.6%, 72.9%, and 69.3%, respectively, of the mean value measured in the control cells (Fig. S3B). Compared with *Mtu1*^*+/−*^ BM-MSCs, Mtu1 knockdown in MS5 cells showed more remarkable alterations in mitochondrial respiratory chain function, ATP synthesis and oxidative stress (Supplementary Figs. S2C–F).

### Alterations in transcriptome analyzed by RNA-seq

**Fig. 6:**
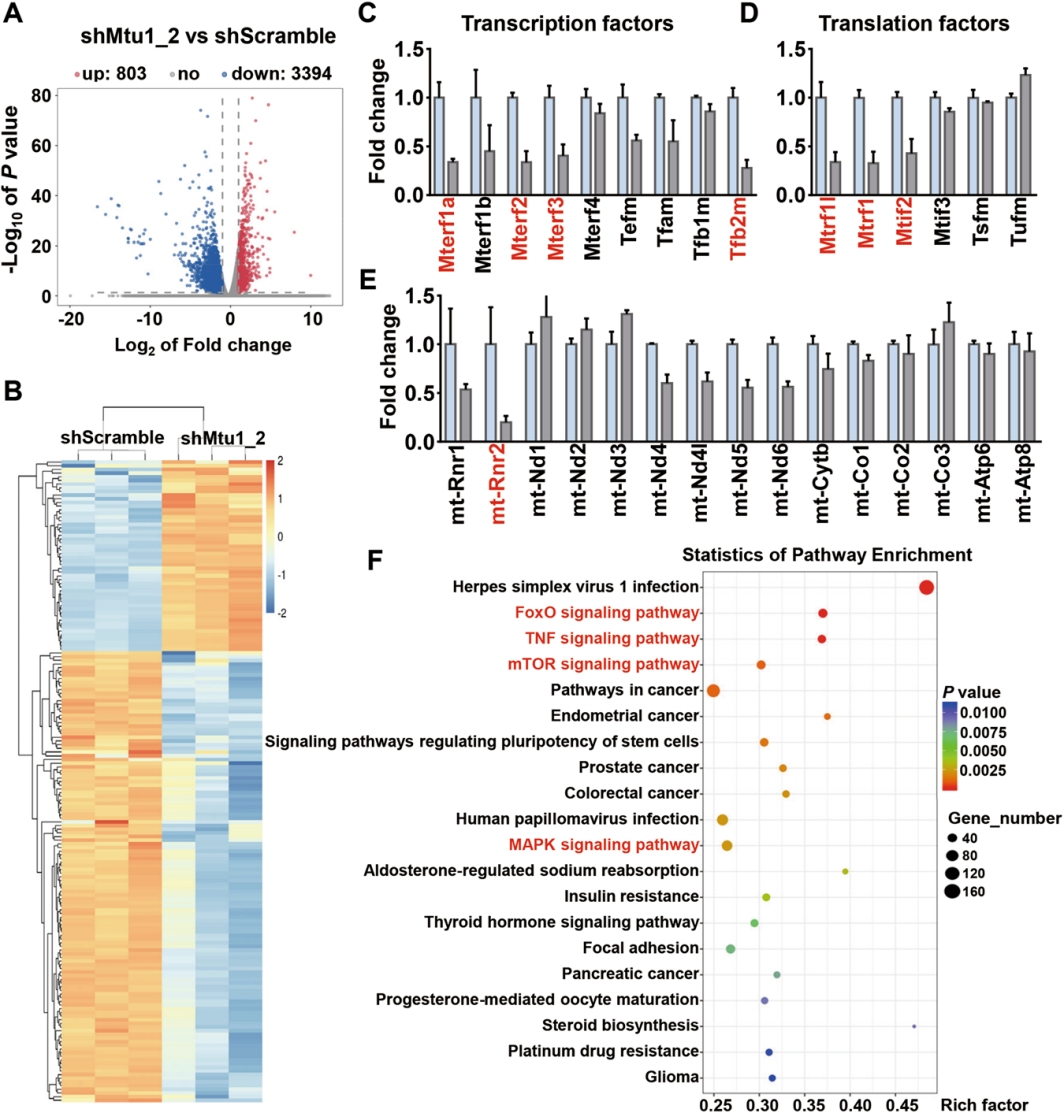
RNA-seq analysis. **A** Volcano plot of differentially expressed genes between shScramble and shMtu1_2 cells. Downregulation and upregulation are shown in blue and red dot, respectively, *n* = 3 per group. **B** Heat map of 174 altered mitochondrial related genes. **C**–**E** The fold changes of mitochondrial transcription factors (**C**), mitochondrial translation factors (**D**), and mitochondrial DNA encoded transcripts (**E**). Genes marked in red color indicate statistically significant (*P* < 0.05) by R package – Ballgown and log_2_ (fold change) > 1 or log_2_ (fold change) < −1. **F** KEGG analyses of RNA-seq data showing the top 20 regulated pathways in Mtu1_2 cells, compared with shScramble cells. *P* values were shown by different color, the size of bubble indicate the gene count of each pathway.

Researchers performed RNA-seq analyses of both shScramble and shMtu1_2 cells to identify the molecular mechanism underlying the MSC osteogenesis differentiation in the Mtu1-deficient group. Compared with shScramble cells, the expression levels of 4197 genes were significantly altered following Mtu1 knockdown (Fig. 6A). Among these differentially expressed genes, 3394 genes were downregulated, and 803 genes were upregulated. By comparing the results to mouse MitoCarta3.0, an inventory of mammalian mitochondrial proteins^36^, 174 genes related to mitochondrial metabolism were found to be statistically different between control and mutant group (Fig. 6B and Supplementary Table S2), including 51 upregulated genes and 123 downregulated genes. The proteins encoded by these altered genes were involved in various mitochondrial metabolisms, including mitochondrial DNA transcription and translation, ribosomal, autophagy, amino acid metabolism and so on (Fig. 6C, D; Supplementary Fig. S3). Meanwhile, there was a downtrend of the mitochondrial DNA encoded transcripts in shMtu1_2 cells, indicating the dysfunction of mitochondrial transcription and translation system in shMtu1_2 cells (Fig. 6E). These results demonstrated that the defect of Mtu1 seriously impacted on mitochondrial biogenesis and metabolism of mesenchymal stem cells.

A biological pathway distribution was observed in KEGG (kyoto encyclopedia of genes and genomes) enrichment analysis (Fig. 6F). There were various signaling pathways influenced by Mtu1 deficiency, including FoxO (forkhead box), TNF (tumor necrosis factor), mTOR, and MAPK (mitogen-activated protein kinase). The signaling pathways were associated with apoptosis, glucose metabolism, oxidative stress resistance, cell proliferation, differentiation, inflammation, immunity and so on. Notably, the FoxO signaling pathway was reported playing key roles in osteoblast differentiation and bone formation^37–39^. Thus, the downregulated FoxO signaling pathway might be the decisive mechanism linking Mtu1 deficiency to dysfunction in osteogenesis.

## Discussion

In the present study, the researchers investigated the roles of Mtu1 in MSC differentiation progress. Mtu1 is a highly conserved mitochondrial enzyme that catalyzes the 2-thiolation of 5-taurinomethyl-2-thiouridine (τm^5^s^2^U) found in the anticodon of a subset of mitochondrial tRNAs (mt-tRNAs), including mitochondrial tRNA^Gln^, tRNA^Glu^ and tRNA^Lys ^^40,41^. Consequently, Mtu1 deficiency resulted in a loss of 2-thiouridylation in mt-tRNAs, leading to a marked mitochondrial translation impairment. Genetic mutations in MTU1 have been identified in reversible infantile liver failure patients and also in hearing loss pedigrees, as the researchers have previously reported. Interestingly, the researchers generated Mtu1 knockout mice and found that these embryonic homozygous-lethal genes failed to develop bone anabolism, and the heterozygous also exhibited symptoms of osteopenia.

Mitochondrial activity or dormancy expectedly plays an important role in maintaining the stemness of MSCs, whereas proper activation is essential for successful differentiation. In fact, τm^5^s^2^U modification is necessary for decoding UUG codons; thus, the deficient modifications at the wobble position of tRNAs led to increased mistranslation^41^. Supporting this speculation, Björk GR et al. showed that a complete loss of the mcm^5^s^2^ modification, the cytosolic counterpart of τm^5^s^2^, resulted in a lethal phenotype in yeast^42^. In this study, the constitutive knockout mice (*Mtu1*^*−∕−*^) were embryonic-lethal at a very early developmental stage (prior to day 8.5).

Compared with *Mtu1*^*−∕−*^ mutants, the phenotypes of *Mtu1*^*+/−*^ mice were rather moderate. Researchers isolated mouse BM-MSCs from the femurs and tibias of *Mtu1*^*+/−*^ and *Mtu1*^*+/+*^ littermates. The inactivation of Mtu1 significantly reduced the 2-thiouridylation in mitochondrial tRNA^Gln^, tRNA^Glu^ and tRNA^Lys^ in *Mtu1*^*+/−*^ BM-MSCs and MS5 stromal cells treated with Mtu1 shRNA. The deficient modifications at wobble position of tRNAs affected the decoding accuracy through altered codon-anticodon interactions and then led to mistranslation^43^. Notably, the decreased steady-state levels of OXPHOS complexes subunits indicated the disruption of mitochondrial translation as well as respiratory chain in Mtu1 deficient cells and consequently resulted in a broad and significant decrease in the activities of Complexes I, III, IV. Furthermore, alterations in mitochondrial translation and respiratory activities led to reduced basal OCR, ATP-linked OCR, reserve capacity and maximal OCR in Mtu1 deficient cells, compared with the controls. The respiratory deficiency then affects the efficiency of mitochondrial ATP synthesis. Several studies have proven that metabolic activity conditions are different between MSCs and differentiated offspring cells^10,12^. The original MSCs are maintained in a niche where they are less dependent on functional mitochondria for energy and that mitochondrial activity needs to increase upon differentiation^44,45^. Interestingly, it was observed that mitochondrial dysfunctions due to Mtu1 deficiency particularly affect the osteogenic differentiation of MSCs. Moreover, RNA-seq analysis revealed that various signaling pathways were dysregulated in MSCs with Mtu1 defects. Among which, the FoxO family of transcription factors were reported to be positive regulator of bone formation and resistance to oxidative stress in osteoblasts^39,46,47^. Thus, downregulation of a large number of genes in FoxO signaling pathway might be a key regulatory mechanism of Mtul related osteogenesis defects.

In summary, this study demonstrated that Mtu1 deficiency caused inefficient 2-thiouridine modification of U34 of mitochondrial tRNA^Gln^, tRNA^Glu^, and tRNA^Lys^ in both primary mouse bone marrow MSCs and MS5 stromal cells, subsequently impairing mitochondrial translation and mitochondrial ATP production, leading to decreased osteogenesis and osteopenia in the mice. These may provide a novel model to study the molecular mechanisms that link mitochondrial dynamics to the osteogenic differentiation regulation.

## Supplementary information

Supplementary Material and Methods

Supplementary Figure Legends

Supplementary Fig. S1

Supplementary Fig. S2

Supplementary Fig. S3

Supplementary Table S1

Supplementary Table S2
