# Supplementary material for: Mtu1 defects are correlated with reduced osteogenic differentiation
Source: Cell Death Dis. 2021 Jan 11;12(1):61. doi: 10.1038/s41419-020-03345-5 (PMC7801634; doi:10.1038/s41419-020-03345-5)
Supplement: Supplementary file 1 — Supplementary Material and Methods [file 41419_2020_3345_MOESM1_ESM.docx]

**Supplementary** **Materials and Methods**

**Mitochondrial tRNA analysis**

The total RNAs were isolated using TRIzol reagent (Ambion Inc.), following the manufacturer’s instructions. Thiouridine modification in the tRNAs was verified by retarding electrophoretic mobility in a 10% polyacrylamide gel that contains 0.05 mg/ml (N-acryloylaminophenyl) mercuric chloride (APM)^1,2^. The RNAs were separated by polyacrylamide gel electrophoresis and electroblotted onto a positively charged membrane (Millipore) for hybridization analysis using oligodeoxynucleotide probes. The sequences of DIG-labeled oligodeoxynucleotide probes were complementary to mouse mitochondrial tRNAs which were consulted from Genomic tRNA Database (GtRNAdb). The tRNA-specific oligodeoxynucleotide probe sequences are shown in Supplementary Table S1. Gel electrophoresis, membrane hybridization, and density quantification in each band were conducted as previously described^3^. Band intensities were quantified from the 16-bit digital image by densitometry in ImageJ.

**Osteogenic differentiation**

Cells were plated in 12 well-plates at a density of 2 $\times$ 10^4^/cm^2^ with 2mL culture medium. Afterwards, the cells stimulated to undergo differentiation were cultured in basal medium supplemented with 10 mM ꞵ-glycerophosphate, 0.1 µM dexamethasone, and 50 µM vitamin C. After 21 days of differentiation, the cells were ﬁxed in 4% paraformaldehyde for 30 min and then washed with PBS, and the mineralized deposits in the matrix were visualized by 0.1% Alizarin Red S after 30 min of staining.

**Adipogenic differentiation**

Cells were plated in 12 well plates at a density of 2 $\times$ 10^4^/cm^2^ on culture dishes with 2mL culture medium. For adipogenic differentiation, the cells were cultured in basal medium containing 10 ng/mL insulin, 1 µM dexamethasone, 0.5 µM 3-isobutyl-1-methylxanthine, and 0.1 mM indomethacin. After 14 days of differentiation, the cells were ﬁxed in 4% paraformaldehyde for 30 min, then washed in 70% isopropanol, and stained with 0.3% Oil Red O for 1 h to visualize lipid vacuoles.

**Mitochondrial respiratory chain enzyme analysis**

Citrate synthase activity was analyzed by the reduction of 5,5′-dithiobis-2-nitrobenzoic acid at 412 nm in the assay buffer containing 0.1 mM DTNB, 50 μM acetyl coenzyme A, and 250 μM oxaloacetate. Complex I activity was determined with 10 μg/ml antimycin A and 2 mM KCN by following the absorbance decrease due to NADH oxidation at 340 nm in the assay buffer. The activity of Complex II was analyzed by tracking the secondary reduction of 2,6-dichlorophenolindophenol by DB at 600 nm in the assay buffer. Complex III activity was determined in the presence of 2 μg/ml antimycin A and 2 mM KCN by measuring cytochrome c reduction at 550 nm with reduced decylubiquinone in the assay buffer. Complex IV activity was measured by monitoring the oxidation of reduced cytochrome c as a decrease of absorbance at 550 nm in the assay buffer. All assays were performed using Synergy H1 (BioTek). Complex I−V activities were normalized by citrate synthase activity^4^.

**Oxygen consumption measurements**

The MSC OCRs were assayed with a Seahorse Bioscience XF-96 extracellular flux analyzer (Seahorse Bioscience), as previously described^5,6^. The protein content of each well was then measured to normalize OCR values.

**Mitochondrial ATP level assessment**

The Cell Titer-Glo® Luminescent Cell Viability Assay kit (Promega) was used for measuring cellular and mitochondrial ATP levels, following the manufacturer’s instructions, with slight modification^5^.

**Mitochondrial ROS production assessment**

MitoSOX assay was used for ROS measurement, following the previously described procedure^7^.

**References**

1 Suzuki, T., Suzuki, T., Wada, T., Saigo, K. & Watanabe, K. Taurine as a constituent of mitochondrial tRNAs: new insights into the functions of taurine and human mitochondrial diseases. *EMBO J.* **21**, 6581-6589 (2002).

2 Meng, F. et al. Biochemical evidence for a nuclear modifier allele (A10S) in TRMU (Methylaminomethyl-2-thiouridylate-methyltransferase) related to mitochondrial tRNA modification in the phenotypic manifestation of deafness-associated 12S rRNA mutation. *J. Biol. Chem.* **292**, 2881-2892 (2017).

3 Li, R. & Guan, M. X. Human mitochondrial leucyl-tRNA synthetase corrects mitochondrial dysfunctions due to the tRNA^Leu(UUR)^ A3243G mutation, associated with mitochondrial encephalomyopathy, lactic acidosis, and stroke-like symptoms and diabetes. *Mol. Cell. Biol.* **30**, 2147-2154 (2010).

4 Spinazzi, M., Casarin, A., Pertegato, V., Salviati, L. & Angelini, C. Assessment of mitochondrial respiratory chain enzymatic activities on tissues and cultured cells. *Nat. Protoc.* **7**, 1235-1246 (2012).

5 Gong, S. et al. A deafness-associated tRNA^His^ mutation alters the mitochondrial function, ROS production and membrane potential. *Nucleic Acids Res.* **42**, 8039-8048 (2014).

6 Dranka, B. P. et al. Assessing bioenergetic function in response to oxidative stress by metabolic profiling. *Free Radic. Biol. Med.* **51**, 1621-1635 (2011).

7 Jia, Z. et al. A coronary artery disease-associated tRNA^Thr^ mutation altered mitochondrial function, apoptosis and angiogenesis. *Nucleic Acids Res.* **47**, 2056-2074 (2019).
