# Supplementary material for: Mtu1 defects are correlated with reduced osteogenic differentiation
Source: Cell Death Dis. 2021 Jan 11;12(1):61. doi: 10.1038/s41419-020-03345-5 (PMC7801634; doi:10.1038/s41419-020-03345-5)
Supplement: Supplementary file 2 — Supplementary Figure Legends [file 41419_2020_3345_MOESM2_ESM.docx]

**Supplementary** **Figure Legends**

**Supplementary Figure S1. Mtu1 knockdown in MS5 did not affect adipogenic differentiation.** A and B. Oil Red O staining and quantification of accumulated lipid droplets in cells after 14 days of adipogenic differentiation. The figures show one representative result of at least 3 experiments. Scale bars: 100 μm. C. RT-qPCR at 0, 7 and 14 days for mRNA levels of osteoblast differentiation markers including *Lpl* and *PPARγ*.

**Supplementary Figure S2. Mitochondrial dysfunction was elevated during osteogenic differentiation.** A. Western blot analysis of subunits of OXPHOS complexes (CI~CV), with Vdac as a loading control. B. The activities of respiratory complexes were investigated by enzymatic assays on complexes of mitochondria isolated from various cell lines. C. Analysis of OCRs in undifferentiated and osteogenesis cell lines using multiple inhibitors. D. Quantification of OCRs revealed significant respiration deficiency. This figure presented the ATP-linked OCR, proton leak OCR, maximal OCR, reserve capacity, and non-mitochondrial OCR in various cell lines. E. Relative ratio of mitochondrial ATP production. F. Mitochondrial ROS production measured by flow cytometry. **P* < 0.05; ***P* < 0.01; ****P* < 0.001.

**Supplementary Figure S3. Heat map of the relative mitochondrial altered genes.** The genes were associated with various mitochondrial metabolisms. The following associated genes were clustered. A. Mitochondrial oxidative phosphorylation (OXPHOS); B. Amino acid metabolism; C. Ribosomal; D. Mitophagy; E. tRNA modification enzymes. All of the genes clustered in Heat map have statistically significant (*P* < 0.5) by R package – Ballgown. Genes marked in red color indicate statistically significant (*P* < 0.05) by R package – Ballgown and log_2_ (fold change) > 1 or log_2_ (fold change) < −1.
