# Supplementary figures and images for: Mtu1 defects are correlated with reduced osteogenic differentiation
Source: Cell Death Dis. 2021 Jan 11;12(1):61. doi: 10.1038/s41419-020-03345-5 (PMC7801634; doi:10.1038/s41419-020-03345-5)

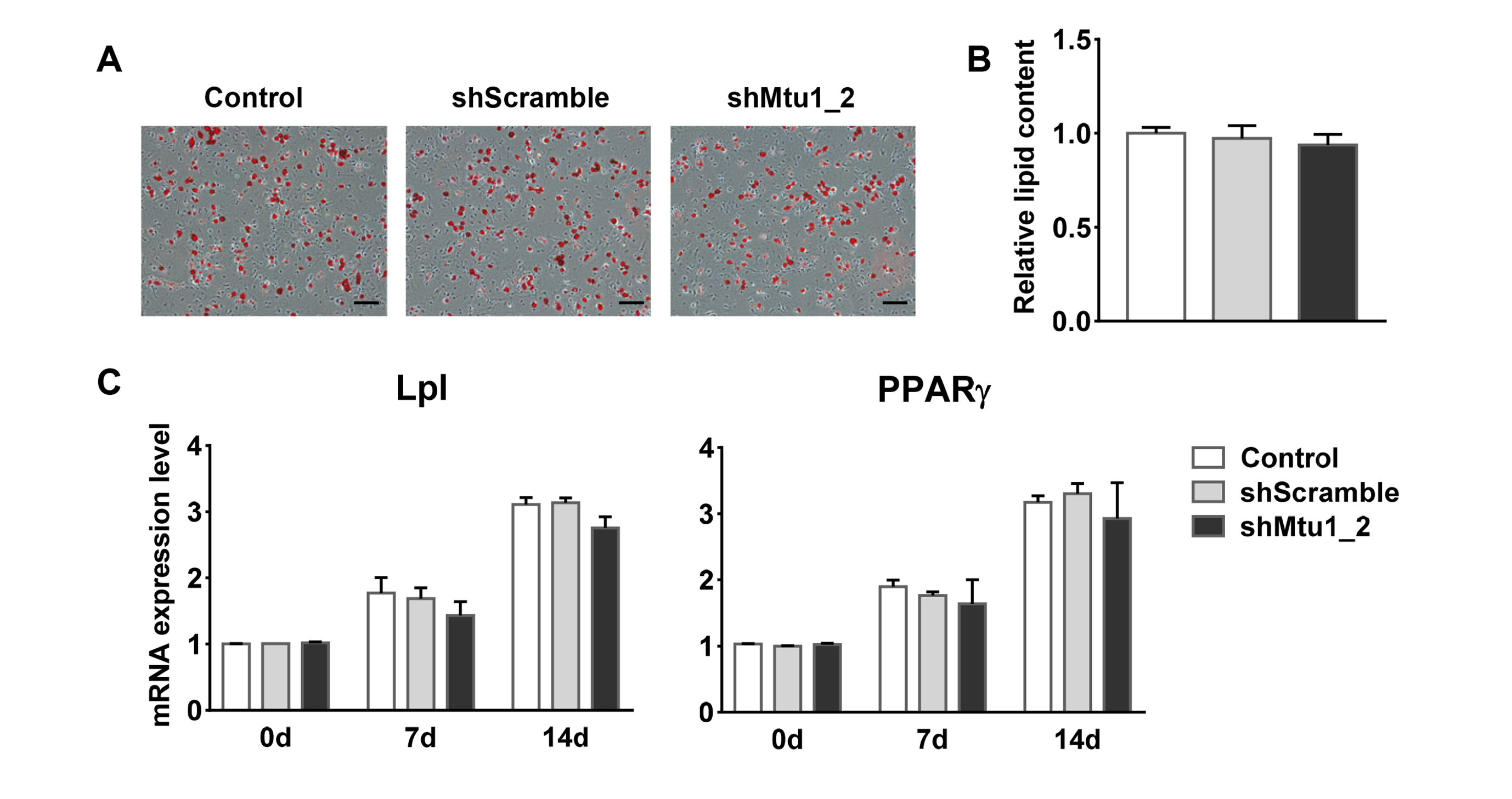

Supplement: Supplementary file 3 — Supplementary Fig. S1 [file 41419_2020_3345_MOESM3_ESM.tif]

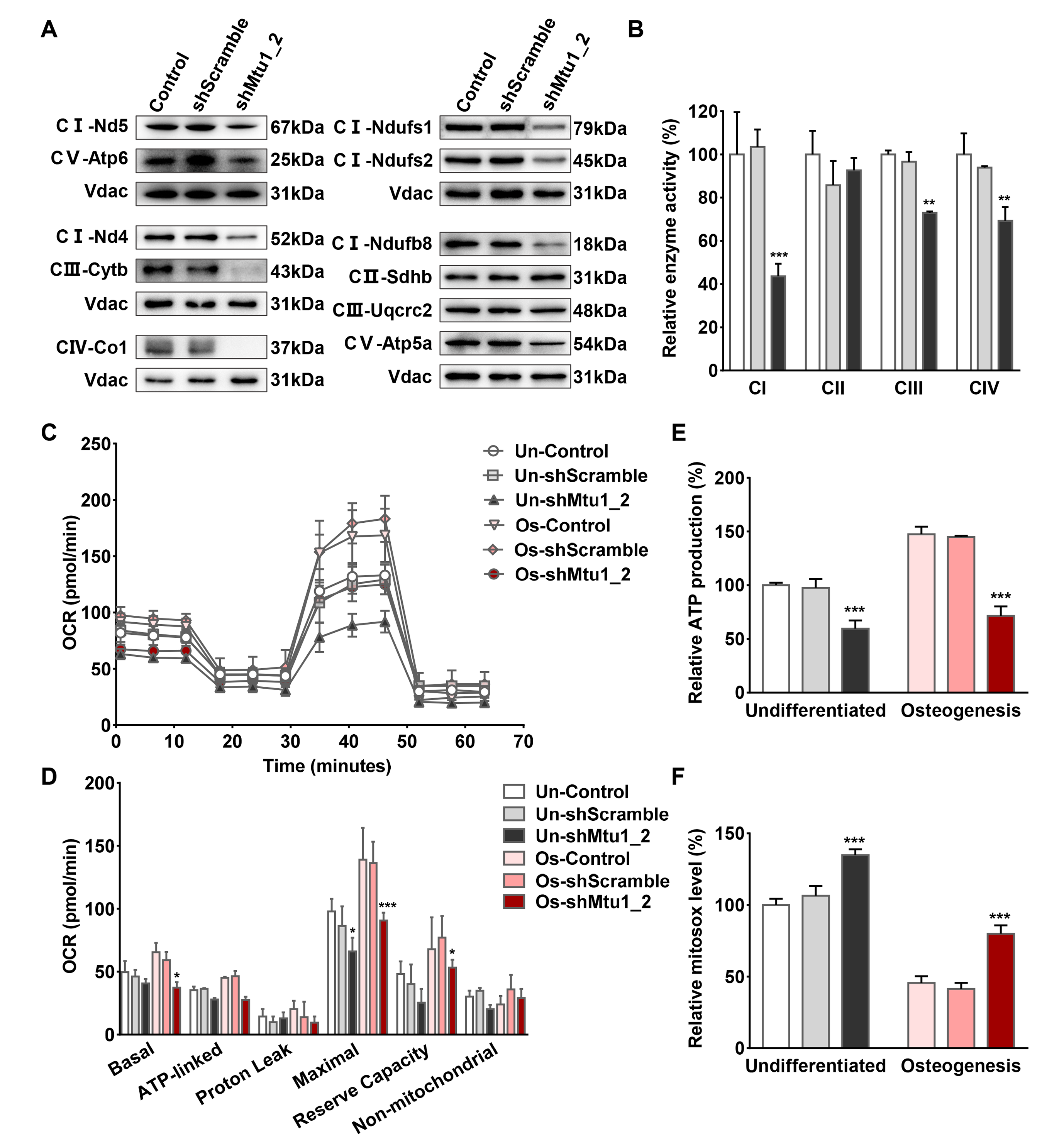

Supplement: Supplementary file 4 — Supplementary Fig. S2 [file 41419_2020_3345_MOESM4_ESM.tif]

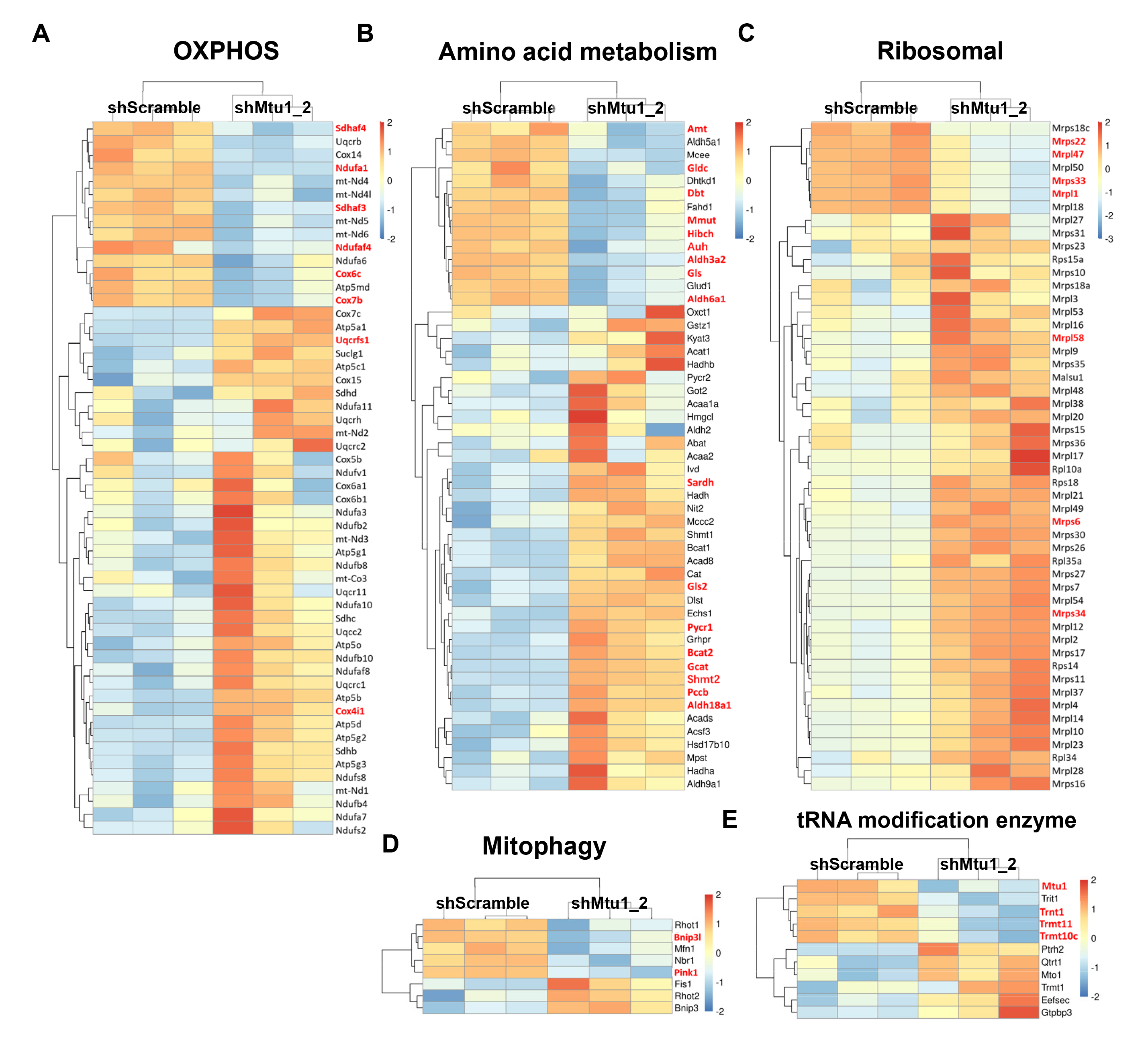

Supplement: Supplementary file 5 — Supplementary Fig. S3 [file 41419_2020_3345_MOESM5_ESM.tif]
