# Supplementary material for: Mtu1 defects are correlated with reduced osteogenic differentiation
Source: Cell Death Dis. 2021 Jan 11;12(1):61. doi: 10.1038/s41419-020-03345-5 (PMC7801634; doi:10.1038/s41419-020-03345-5)
Supplement: Supplementary file 6 — Supplementary Table S1 [file 41419_2020_3345_MOESM6_ESM.docx]

1. **Sequences of insertions encoding shRNA.**

| **shRNA** | **Oligonucleotides Sequence (5’~3')** |
| --- | --- |
| shScramble | CCGGAAGCTTCGCGCCGTAGTCTTACTCGAGTAAGACTACGGCGCGAAGCTTTTTTTG |
| shMtu1_1 | CCGGTTTGTTTCTATCGAGGATAATCTCGAGATTATCCTCGATAGAAACAAATTTTTG |
| shMtu1_2 | CCGGGACTGTGAAGATGCGTATAAACTCGAGTTTATACGCATCTTCACAGTCTTTTTG |

1. **tRNA-specific oligodeoxynucleotide probes.**

| **tRNA** | **5' DIG-oligodeoxynucleoside probe (5’~3')** |
| --- | --- |
| tRNA^Gln^ | CTAGGACAATAGGAATTGAACCTACACTTA |
| tRNA^Glu^ | TATTTCTACACAGCATTCAACTGCGACCAA |
| tRNA^Lys^ | TCACTATGGAGATTTTAAGGTCTCTAACTT |
| tRNA^Leu(UUR)^ | TATTAGGGAGAGGATTTGAACCTCTGGGAA |

1. **Sequences of oligodeoxynucleosides for real-time PCR analysis.**

| **Gene** | **Forward primer (5’~3')** | **Reverse primer (5’~3')** |
| --- | --- | --- |
| Runx2 | CCGCACGACAACCGCACCAT | CGCTCCGGCCCACAAATCTC |
| Alp | GCCCTCTCCAAGACATATA | CCATGATCACGTCGATATCC |
| Ocn | AAGCAGGAGGGCAATAAGGT | AGCTGCTGTGACATCCATAC |
| Lpl | GGGCTCTGCCTGAGTTGTAG | AGAAATTTCGAAGGCCTGGT |
| Pparγ | CCCTGGCAAAGCATTTGTAT | AATCCTTGGCCCTCTGAGAT |
| Gapdh | TCAACAGCAACTCCCACTCTTCCA | ACCCTGTTGCTGTAGCCGTATTCA |
